# Predictive value of the C-reactive protein–triglyceride–glucose index for late atrial fibrillation recurrence after radiofrequency catheter ablation: a retrospective cohort study

**DOI:** 10.1186/s12872-026-06238-1

**Published:** 2026-07-22

**Authors:** Su-Yuan He, Ming-Hu Zhao, Yuan Qin, Ju-Gang Chen

**Affiliations:** 1 Department of Cardiology, Henan Provincial People’s Hospital, People’s Hospital of Zhengzhou University, Zhengzhou, Henan 450003 China; 2 Department of Plastic and aesthetic surgery, Henan Provincial People’s Hospital, People’s Hospital of Zhengzhou University, Zhengzhou, 450003 China

**Keywords:** Atrial fibrillation, CTI, Late recurrence, Radiofrequency catheter ablation

## Abstract

**Background:**

The C-reactive protein–triglyceride–glucose index (CTI) has been reported to be positively associated with cardiovascular disease, stroke, and other adverse outcomes. However, the association between CTI and late atrial fibrillation (AF) recurrence after radiofrequency catheter ablation (RFCA) has not been well established.

**Methods:**

A total of 591 patients with AF who underwent RFCA were retrospectively enrolled. CTI was calculated using triglycerides, fasting plasma glucose, and C-reactive protein. The primary endpoint was late AF recurrence during postoperative follow-up. The association between CTI and late AF recurrence after RFCA was assessed using restricted cubic spline (RCS) analysis and univariate and multivariable logistic regression analyses. Receiver operating characteristic curve analysis and the DeLong test were conducted to assess the predictive performance of CTI. Net reclassification improvement (NRI) and integrated discrimination improvement (IDI) were adopted to evaluate its incremental predictive value. The Hosmer–Lemeshow test and Bootstrap-calibrated curves were used to verify model calibration and goodness-of-fit. Subgroup analyses were conducted to examine potential variations in the association between CTI and AF recurrence across subgroups. Subgroup analyses were conducted to examine potential variations in the association between CTI and AF recurrence across subgroups.

**Results:**

All patients were followed for 1 year postoperatively, during which 92 cases of AF recurrence were documented. Higher CTI levels were positively associated with an increased risk of AF recurrence. RCS analysis suggested that the nonlinear test did not reach statistical significance (p for non-linear = 0.072), while a trend toward nonlinearity was observed, which may be explained by the limited number of outcome events. After multivariable adjustment, CTI remained independently and positively associated with AF recurrence, with an odds ratio of 6.20 per one-unit increase in CTI (95% CI: 3.56–10.77; *p* < 0.001). Subgroup and interaction analyses yielded findings consistent with the primary results.

**Conclusions:**

CTI was identified as a potentially valuable predictive indicator for late AF recurrence following RFCA.

**Supplementary Information:**

The online version contains supplementary material available at https://doi.org/10.1186/s12872-026-06238-1.

## Background

Atrial fibrillation (AF) is the most prevalent progressive arrhythmia worldwide, which is a major contributor to elevated risks of stroke, heart failure, peripheral vascular embolism, and other adverse outcomes [1]. Multiple randomized controlled trials have demonstrated that radiofrequency catheter ablation (RFCA) was superior to antiarrhythmic drug therapy in maintaining sinus rhythm [2]. Postoperative AF recurrence remains a significant clinical challenge, with reported recurrence rates ranging from 30% to 60% among patients undergoing the procedure, despite substantial technological advancements in RFCA in recent years [3]. There are numerous risk factors associated with atrial AF recurrence after RFCA. Although previous studies have confirmed that several existing prediction models, such as the APPLE score and CAAP-AF score, possess certain predictive value for AF recurrence following RFCA, the predictive accuracy of these scores remains limited. In a systematic review focusing on the prediction of AF recurrence after RFCA, Janine D et al. included 13 prediction models for AF recurrence after RFCA, including the APPLE score and CAAP-AF score, and found that none of these models exhibited consistently excellent discriminative ability across the studies [4]. Additionally, the calculation process of these models is relatively complex, and the medical history information required for some scores mostly relies on self-reporting by patients, which is prone to interference from patients’subjective factors. Therefore, there is an urgent clinical need for an objective and easy-to-calculate indicator to assess the recurrence risk of AF in patients after RFCA.

AF represents a pathophysiological continuum in which atrial remodeling serves as a central substrate for both initiation and perpetuation of the arrythmia [5]. Insulin resistance (IR), a mechanistic link between metabolic syndrome and glucose dysregulation, was associated with left atrial remodeling and contributes to the initiation and maintenance of AF through multiple converging pathways [6, 7]. In addition, inflammation plays a key role in the pathogenesis of AF by promoting atrial remodeling and facilitating arrhythmia onset [8].

The triglyceride–glucose (TyG) index, derived from fasting triglyceride and glucose levels, has been widely used as a surrogate marker of IR [9, 10]. When compared to gold-standard methods, including the hyperinsulinemic–euglycemic clamp and homeostasis model assessment of IR (HOMA-IR), it provides a cost-effective and readily accessible approach for assessing insulin sensitivity and predicting the risk of metabolic and cardiovascular disorders [9, 11]. Prior studies have reported associations between the TyG index and the pathophysiological mechanisms underlying AF and its recurrence [9]. Therefore, as a noninvasive and clinically practical marker, the TyG index may assist in identifying patients at increased risk of AF recurrence after RFCA [12]. As a nonspecific marker of inflammation, elevated C-reactive protein (CRP) levels have been reported to be associated with AF persistence and recurrence following radiofrequency ablation [13]. Overall, inflammation and IR are considered to adversely influence AF recurrence after radiofrequency ablation. However, prior studies primarily examined the independent associations of the TyG index and CRP with AF recurrence after RFCA, and data regarding their combined effect remain limited. The C-reactive protein–triglyceride–glucose index (CTI), an integrated biomarker reflecting both inflammation and insulin resistance, was significantly associated with the risk of cardiovascular disease (CVD) [14, 15]. However, the association between CTI and AF recurrence after RFCA remains unclear. Therefore, this study aimed to assess the predictive utility of CTI for 1-year AF recurrence after RFCA in patients with AF and to provide evidence to inform preprocedural risk stratification and individualized clinical management.

## Methods

### Study design and participants

**Fig. 1:**
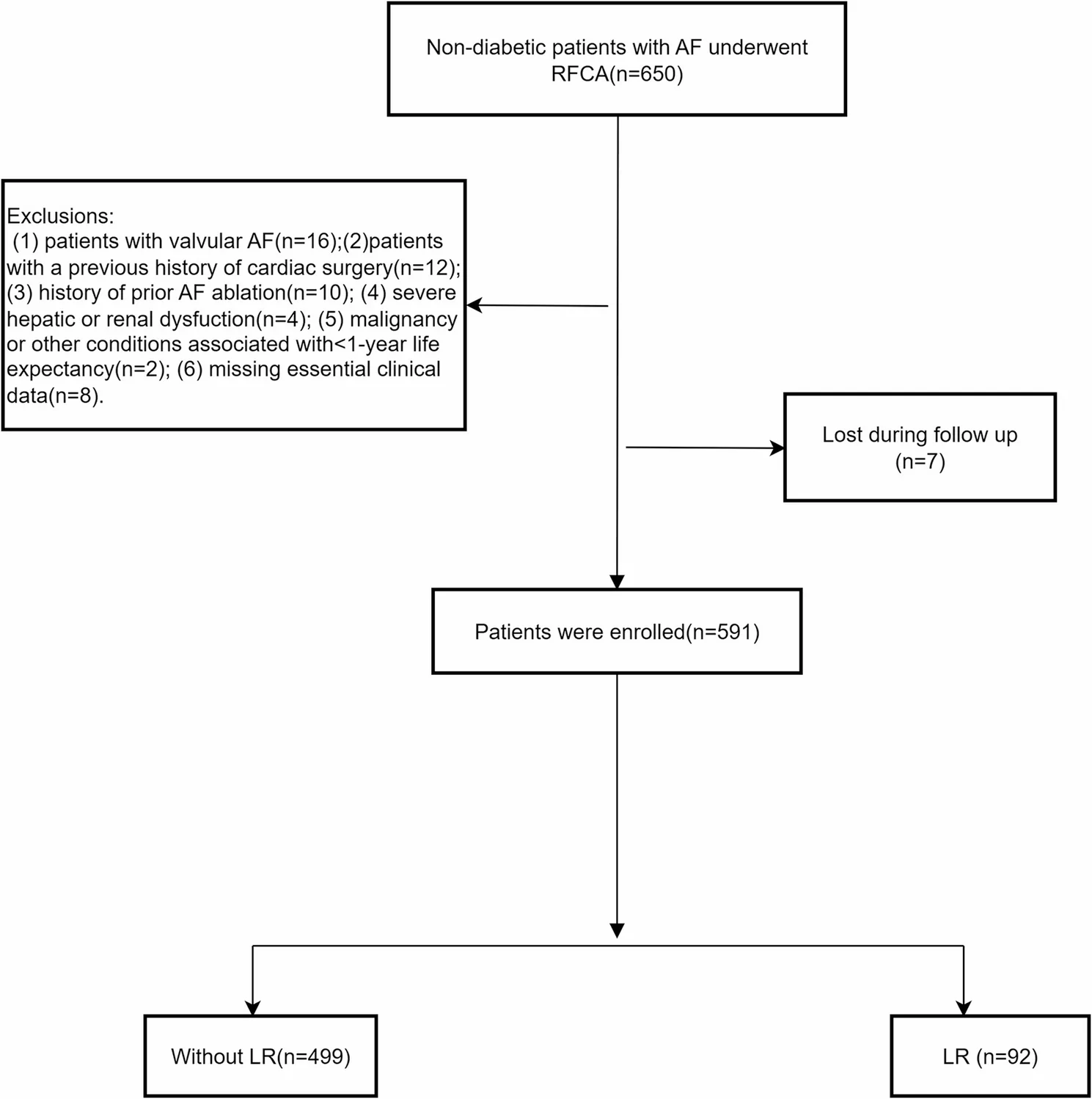
Flow diagram of participant selection. (AF, atrial fibrillation; RFCA, radiofrequency catheter ablation; LR, late recurrence; Without LR, without late recurrence)

A retrospective, single-center observational study was conducted among patients with non-valvular AF who presented to Henan Provincial People’s Hospital between January 2020 and December 2024. All included patients underwent RFCA during hospitalization. Exclusion criteria were as follows: (1) valvular AF; (2) a history of cardiac surgery; (3) prior AF ablation; (4) severe hepatic or renal dysfunction; (5) malignancy or other conditions associated with a life expectancy of less than 1 year; and (6) missing essential clinical data (Fig. 1). The study was approved by the Ethics Review Committee of Henan Provincial People’s Hospital (No. 2024-59) and was conducted in accordance with the Declaration of Helsinki.

### Data collection and definitions

Data were extracted from the electronic medical record systems of the participating healthcare institution by trained investigators. The dataset comprised demographic and clinical characteristics, laboratory results, echocardiographic parameters, and prior outpatient medication use. Specifically, data were collected on age, sex, body mass index (BMI), AF type, laboratory test results, comorbidities (hypertension, diabetes mellitus (DM), coronary heart disease (CHD), heart failure, and stroke), echocardiographic parameters, and prior medications, including antiarrhythmic drugs, sodium–glucose cotransporter 2 inhibitors (SGLT-2 inhibitors), anticoagulant drugs and angiotensin receptor–neprilysin inhibitors.

Fasting peripheral venous blood samples were obtained for measurement of CRP, fasting blood glucose (FBG), triglycerides (TG), total cholesterol (TC), low-density lipoprotein cholesterol, high-density lipoprotein cholesterol (HDL-C), hemoglobin, and N-terminal pro-B-type natriuretic peptide (NT-proBNP). Transthoracic echocardiography was conducted to assess left atrial diameter (LAD) and left ventricular ejection fraction (LVEF). Comorbidities, including hypertension, DM, and CHD, were defined in accordance with established clinical diagnostic criteria [16].

Persistent AF was defined as AF lasting more than 7 days, whereas paroxysmal AF was defined as AF that terminated spontaneously or following pharmacological or electrical cardioversion within 7 days [17]. CTI was calculated using CRP, FBG, and TG according to the following formula:$0.412\times\mathrm{Ln}(\mathrm{CRP}\lbrack\mathrm{mg}/\mathrm L\rbrack)+\mathrm{Ln}(\mathrm{TG}\lbrack\mathrm{mg}/\mathrm{dL}\rbrack\times\mathrm{FBG}\lbrack\mathrm{mg}/\mathrm{dL}\rbrack)/2$ [18].

### Catheter ablation strategies and methods

All patients underwent RFCA under general anesthesia. A three-dimensional anatomical reconstruction of the left atrium was generated using the CARTO 3 (Three-Dimensional Electroanatomical Mapping System; Biosense Webster Inc., USA). The PentaRay catheter was advanced into the left atrium to perform baseline mapping.

Circular pulmonary vein isolation (CPVI) was performed in all patients with paroxysmal AF and non-paroxysmal AF (NPAF). In patients with NPAF, additional ablation strategies were implemented beyond CPVI when clinically indicated. The ablation sequence included the following: Marshall vein alcohol ablation; left atrial apical line ablation; mitral isthmus ablation (linear catheter ablation across the mitral isthmus was initially performed in all patients; if bidirectional complete conduction block at the mitral isthmus was not achieved intraoperatively, epicardial key point ablation was additionally performed); superior vena cava ablation; and tricuspid isthmus ablation. If AF persisted after completion of the ablation procedures, intravenous ibutilide (1 mg) was administered or electrical cardioversion was performed to restore sinus rhythm.

### Study outcome

All patients received antiarrhythmic drugs (AADs) for 3 months following radiofrequency ablation, unless contraindications to AADs were present, for the prevention of early AF recurrence. Routine outpatient follow-up visits were scheduled at 3, 6, and 12 months postoperatively. At each visit, medical history was obtained, physical examination was performed, and 12-lead electrocardiography (ECG), 24-hour ambulatory ECG monitoring, or long-term ambulatory ECG monitoring was conducted. The primary endpoint was late AF recurrence during follow-up after the 3-month blanking period following the initial RFCA. Late recurrence was defined as any documented episode of AF or atrial tachycardia lasting ≥ 30 s, as confirmed by standard ECG or ambulatory ECG monitoring, occurring after the 3-month blanking period.

### Statistical analysis

Continuous variables with a normal distribution were expressed as mean ± standard deviation. Comparisons between two groups were performed using the independent-samples t-test, and comparisons among multiple groups were conducted using one-way analysis of variance. For data that were not normally distributed, values were presented as median [P25, P75]. Intergroup comparisons were conducted using the Mann–Whitney U test for two groups or the Kruskal–Wallis H test for multiple groups. Categorical variables were expressed as the number of cases (percentage, n/%). Comparisons between groups were conducted using the chi-square test or Fisher’s exact test, as appropriate.

Recurrence rates across different CTI levels, categorized by tertiles, were illustrated using bar charts, and the chi-square test for trend was performed to assess the association between CTI levels and recurrence risk. Restricted cubic spline (RCS) curves were generated using the rms package in R software to assess potential nonlinear associations between continuous CTI values and the outcome, with the median CTI value designated as the reference. The model was adjusted for relevant covariates, including sex, age, BMI, type of AF, stroke, amiodarone use, NT-proBNP, HDL, LAD, and LVEF. In addition, univariate and multivariable logistic regression analyses were conducted to examine the association between CTI levels (analyzed both as a continuous variable and as tertiles based on CTI values) and the risk of the outcome. Multiple regression models were constructed: Model 1 was unadjusted; Model 2 was adjusted for age, sex, and BMI; and Model 3 was adjusted for all variables included in Model 2, with additional adjustment for type of AF, stroke, amiodarone use, NT-proBNP, HDL, LAD, and LVEF. A test for trend across tertile groups was performed using the median CTI value of each tertile.

Receiver operating characteristic (ROC) curve analysis was conducted to estimate the area under the curve (AUC) and corresponding 95% confidence interval (95% CI) for CTI in predicting the outcome, thereby assessing its predictive performance. The DeLong test was applied to compare the predictive value of different indicators, and clinical decision curve analysis (DCA) was further conducted to assess their comparative clinical use. Net reclassification improvement (NRI) and integrated discrimination improvement (IDI) were further applied to evaluate the incremental predictive value of CTI relative to conventional indicators. Meanwhile, the Hosmer–Lemeshow (HL) test and Bootstrap‑calibrated calibration curves were used to assess model goodness-of-fit and probability calibration.

To further assess whether the association between CTI and the outcome differed across populations with varying characteristics, subgroup analyses were conducted. In these analyses, covariate adjustments were consistent with those applied in Model 3, apart from the respective subgroup variable. The results of the subgroup analyses were presented using forest plots.

All statistical analyses and corresponding graphical representations were performed using R software (version 4.4.1). A two-tailed *p* < 0.05 was considered to indicate statistical significance.

## Results

### Baseline characteristics

A total of 591 patients were included in the final analysis between January 2020 and December 2024, of whom 61.6% were male. The mean age was 62.55 ± 10.57 years. Patients were stratified into three groups according to tertiles of CTI, with 197 patients in each group. The mean CTI values in the three groups were 4.17 ± 0.15, 4.55 ± 0.11, and 5.17 ± 0.31; respectively (Table 1; Supplementary Table 1).

**Table 1:** Baseline characteristics of patients stratified by atrial fibrillation recurrence

Characteristics	Total (*n* = 591)	AF recurrence (-) (*n* = 499)	AF recurrence (+) (*n* = 92)	*p* value
Demographics				
Male, n(%)	364 (61.6)	307 (61.5)	57 (62)	0.937
Age, years, Mean ± SD	62.55 ± 10.57	62.21 ± 10.51	64.41 ± 10.74	0.066
BMI, kg/m^2^, Mean ± SD	25.66 ± 3.85	25.43 ± 3.68	26.89 ± 4.53	< 0.001
Medical history				
NPAF, n(%)	251 (42.5)	195 (39.1)	56 (60.9)	< 0.001
Hypertension, n (%)	261 (44.2)	216 (43.3)	45 (48.9)	0.318
Diabetes mellitus (%)	95 (16.1)	79 (15.8)	16 (17.4)	0.708
Coronary heart disease (%)	65 (11)	55 (11)	10 (10.9)	0.966
Congestive heart failure, n(%)	77 (13)	64 (12.8)	13 (14.1)	0.733
Stroke (%)	73 (12.4)	69 (13.8)	4 (4.3)	0.011
Laboratory data				
NT-proBNP, pg/mL, Median (IQR)	436(148,1010)	392 (125.5, 969)	599 (312.5, 1425)	0.002
Cr, µmol/L, Median (IQR)	67 (56, 78.65)	67 (56, 79)	66 (58, 76.88)	0.728
TC, mmol/L, Median (IQR)	3.88 (3.28, 4.50)	3.89 (3.29, 4.51)	3.83 (3.27, 4.43)	0.535
LDL, mmol/L, Median (IQR)	2.16 (1.66, 2.65)	2.14 (1.67, 2.65)	2.23 (1.65, 2.60)	0.844
HDL, mmol/L, Median (IQR)	1.08 (0.93, 1.28)	1.09 (0.95, 1.30)	1.03 (0.85, 1.20)	0.005
HGB, g/L, Mean ± SD	139.37 ± 16.99	139.57 ± 16.55	138.30 ± 19.28	0.557
FBG, mg/dl, Mean ± SD	107.90 ± 31.66	106.60 ± 31.36	114.94 ± 32.55	0.020
TG, mg/dL, Mean ± SD	129.69 ± 73.29	125.66 ± 70.40	151.58 ± 84.46	0.007
TyG, Mean ± SD	8.70 ± 0.55	8.66 ± 0.54	8.92 ± 0.57	< 0.001
CRP, mg/L, Mean ± SD	1.29 ± 1.49	1.08 ± 1.29	2.41 ± 1.95	< 0.001
CTI, Mean ± SD	4.63 ± 0.46	4.56 ± 0.42	5.00 ± 0.47	< 0.001
UCG				
LAD, Median (IQR)	42 (36, 46)	41(36, 45)	45 (40, 48)	< 0.001
LVEF, Median (IQR)	63 (59, 67)	63(59, 67)	61 (56, 66)	0.011
Drug history				
amiodarone	31 (5.2)	19 (3.8)	12 (13)	< 0.001
ARNI	82 (13.9)	68 (13.6)	14 (15.2)	0.685
SGLT-2	44 (7.4)	36 (7.2)	8 (8.7)	0.619
Anticoagulant Drugs	256 (43.3)	206 (41.3)	50 (54.3)	0.020
Predictive Model Score				
APPLE score, Mean ± SD	1.42 ± 1.08	1.33 ± 1.04	1.90 ± 1.15	< 0.001
CAAP-AF score, Mean ± SD	4.20 ± 2.17	4.02 ± 2.11	5.17 ± 2.22	< 0.001

Data were expressed as mean ± SD, median with 25th and 75th percentile or n (%)

*BMI* Body mass index; AF: atrial fibrillation, *NPAF* Nonparoxysmal atrial fibrillation, *NT-pro-BNP* N-terminal pro-B-type natriuretic peptide, *Cr* creatinine, *TC* total cholesterol, *LDL-C* Low-density lipoprotein cholesterol, *HDL-C* High-density lipoprotein cholesterol, *HGB* Hemoglobin, *FBG* Fasting blood glucose, *TG* Triglyceride, *TyG* Triglyceride glucose index, *CRP* C-reactive protein-Triglyceride, *CTI* C-reactive protein-Triglyceride-Glucose Index, *LAD* left atrial diameter, *LVEF* Left ventricular ejection fraction, *ARNI* Angiotensin Receptor-Neprilysin Inhibitor, *SGLT2* Sodium-Glucose Cotransporter 2, *APPLE score* Included variables were age, type of AF, eGFR, LAD, and LVEF, *CAAP-AF score* Included variables were age, sex, type of AF, LAD, coronary artery disease, and number of antiarrhythmic drugs failed

During the 1-year follow-up period, AF recurrence occurred in 92 patients. Compared with patients without recurrence, those with AF recurrence were older and had a higher prevalence of persistent AF. In addition, BMI, NT-proBNP, FBG, TG, and CRP levels were significantly higher in the recurrence group (all *p* < 0.05). LAD was significantly greater in patients with recurrence than in those without recurrence (*p* < 0.001).

### Relationship between CTI and AF recurrence

**Fig. 2:**
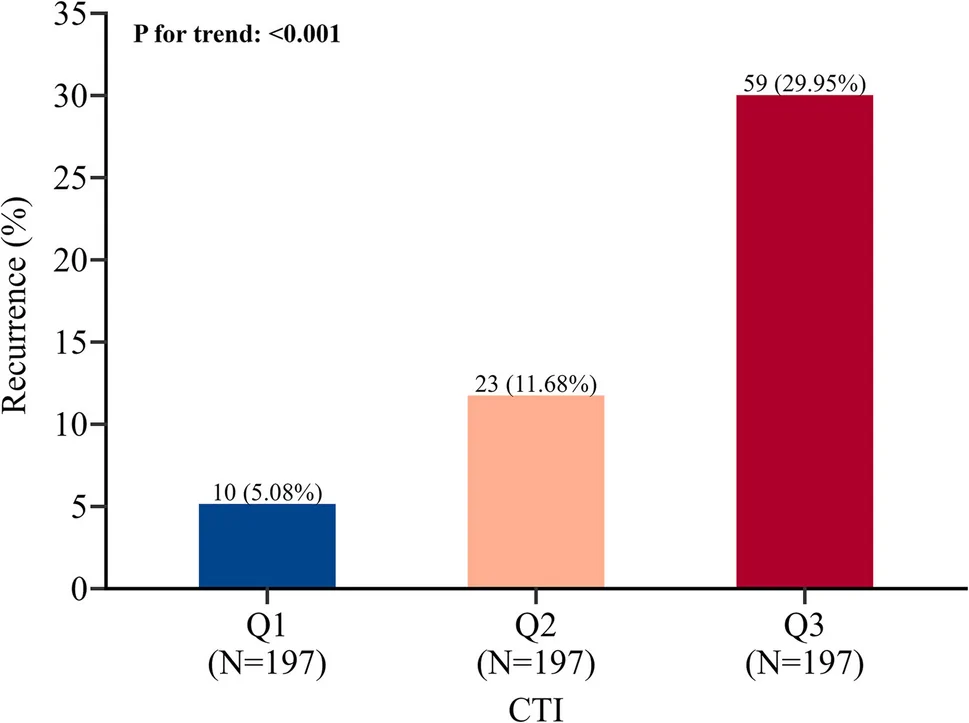
Comparison of atrial fibrillation (AF) recurrence rates across CTI tertile groups

**Fig. 3:**
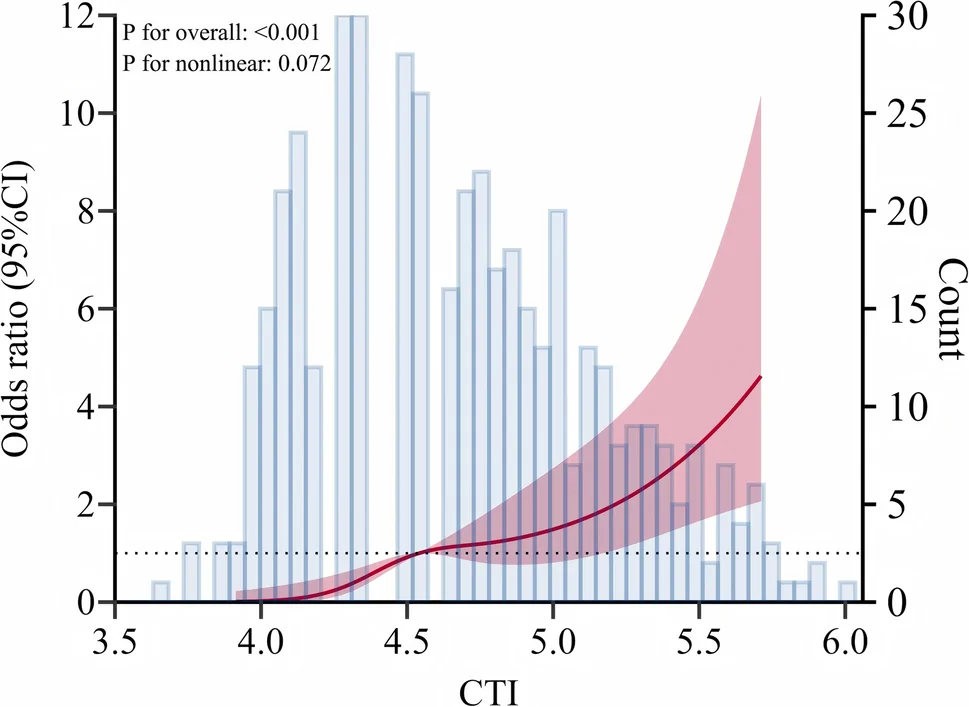
Association between CTI and AF recurrence assessed using RCS analysis. The threshold for statistical significance was set at *p* < 0.05. Odds ratios are presented as solid lines, and 95% CIs are displayed as shaded areas. (OR, Odds ratio; CI, confidence interval; CTI, C-reactive protein-triglyceride glucose index)

During the follow-up period, AF recurrence was documented in 92 patients. As presented in Fig. 2, the number of events and AF recurrence rates were presented according to CTI tertiles. The recurrence rates in the Q1, Q2, and Q3 groups were 5.08%, 11.68%, and 29.95%, respectively. AF recurrence rates in the Q2 and Q3 groups were significantly higher than those in the Q1 group, with a progressive increase observed across increasing CTI tertiles. A positive association was observed between CTI levels and AF recurrence rates; the recurrence rate in the highest CTI group (Q3) was approximately sixfold higher than that in the lowest group (Q1) (Fig. 2). RCS analysis showed that CTI was significantly associated with AF recurrence overall (P for overall < 0.001). The spline curve suggested a possible nonlinear trend, with the risk of recurrence tending to increase at higher CTI levels; however, the test for nonlinearity did not reach statistical significance (P for nonlinearity = 0.072).The curve shows that the OR of AF recurrence gradually increases when CTI exceeds 4.5, revealing a tentative threshold-like trend. Although this CTI cutoff of 4.5 cannot serve as a definitive threshold for clinical intervention, it provides a potential direction for future external validation research (Fig. 3).

Univariate and multivariable logistic regression analyses demonstrated that higher CTI values were positively associated with an increased risk of AF recurrence.

In Model 1 (unadjusted for confounding factors), patients in the second and third tertile groups exhibited a higher risk of AF recurrence compared with those in the first tertile group. Specifically, patients in the third tertile group had a 7.99-fold higher risk of AF recurrence than those in the first tertile group (95% CI: 3.95–16.19; *p* < 0.001). For each one-unit increase in CTI, the odds ratio (OR) for AF recurrence was 7.16 (95% CI: 4.32–11.87; *p* < 0.001).

**Fig. 4:**
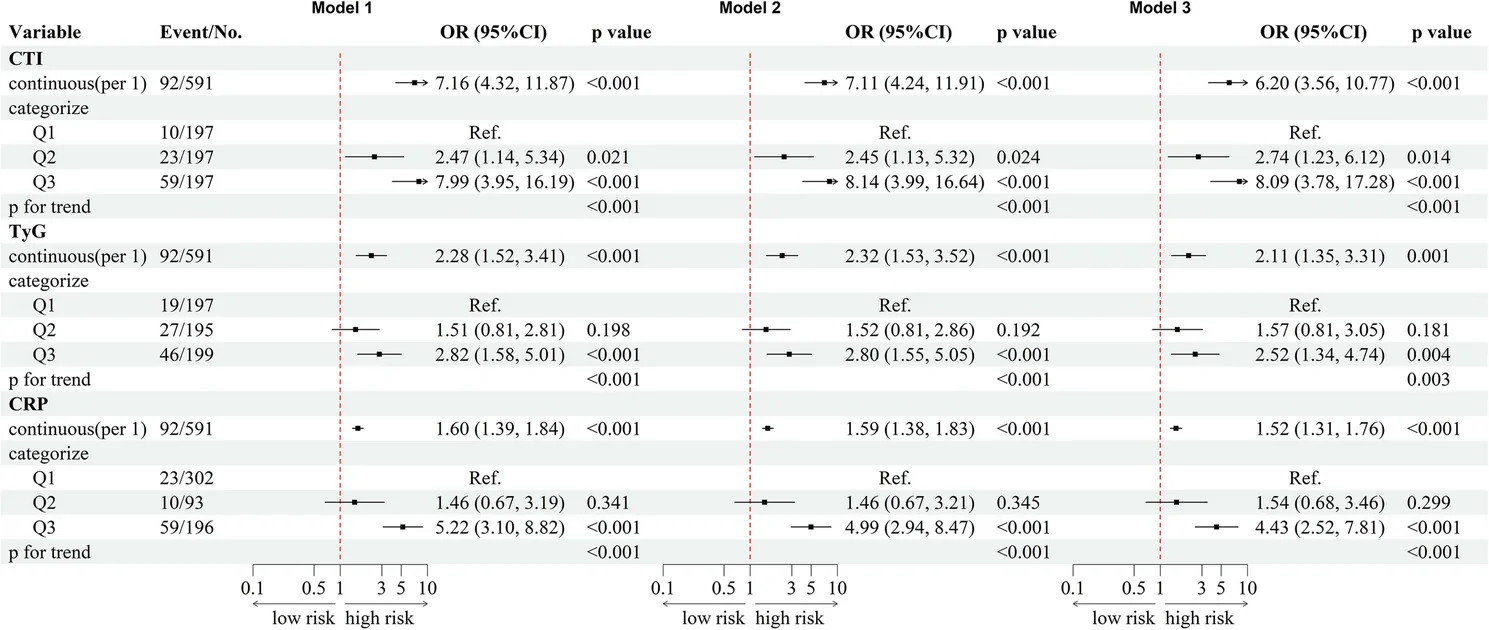
Forest plot of univariate and multivariable logistic regression analyses examining the association between CTI and AF recurrence. OR, Odds ratio; CI, confidence interval; Q1/Q2/Q3, CTI tertiles. Model 1 was unadjusted. Model 2 was adjusted for age, sex, and BMI. Model 3 was further adjusted for AF type, stroke, amiodarone use, NT-pro B-type natriuretic peptide (BNP), high-density lipoprotein (HDL), left atrial diameter (LAD), and left ventricular ejection fraction (LVEF), along with the variables included in Model 2. (CTI, C-reactive protein-triglyceride glucose index; TyG, Triglyceride-Glucose; CRP, C-reactive protein; OR, Odds Ratio)

In Model 2 (adjusted for age, sex, and BMI), this association remained consistent. Patients in the third tertile group had an 8.14-fold higher risk of AF recurrence compared with those in the first tertile group (95% CI: 3.99–16.64; *p* < 0.001). For each one-unit increase in CTI, the OR for AF recurrence was 7.11 (95% CI: 4.24–11.91; *p* < 0.001). After further adjustment for all variables included in Model 2, along with the type of AF, stroke, AAD use, NT-proBNP, HDL, LAD, and LVEF (Model 3), the association remained significant. Patients in the third tertile group had an 8.09-fold higher risk of AF recurrence compared with those in the first tertile group (95% CI: 3.78–17.28; *p* < 0.001). In addition, each one-unit increase in CTI was associated with an OR of 6.20 for AF recurrence (95% CI: 3.56–10.77; *p* < 0.001) (Fig. 4).

### Use of CTI in predicting AF recurrence

**Fig. 5:**
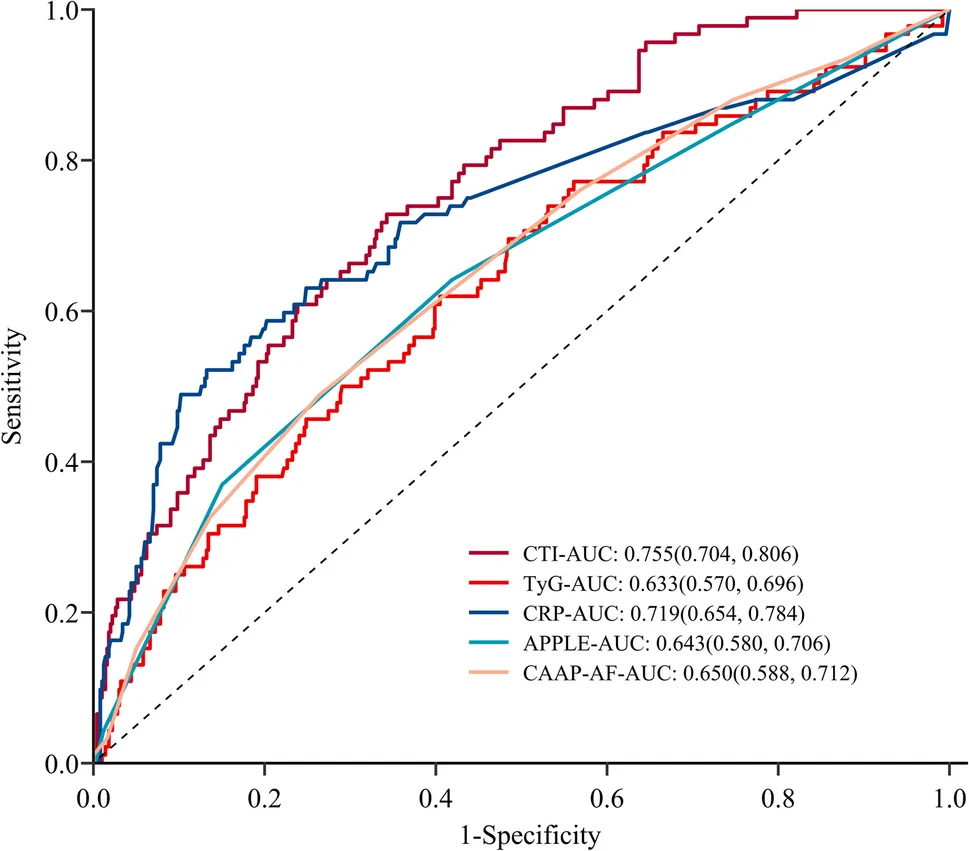
Predictive performance of CTI for AF recurrence. ROC curve analysis; AUC, area under the ROC curve

**Fig. 6:**
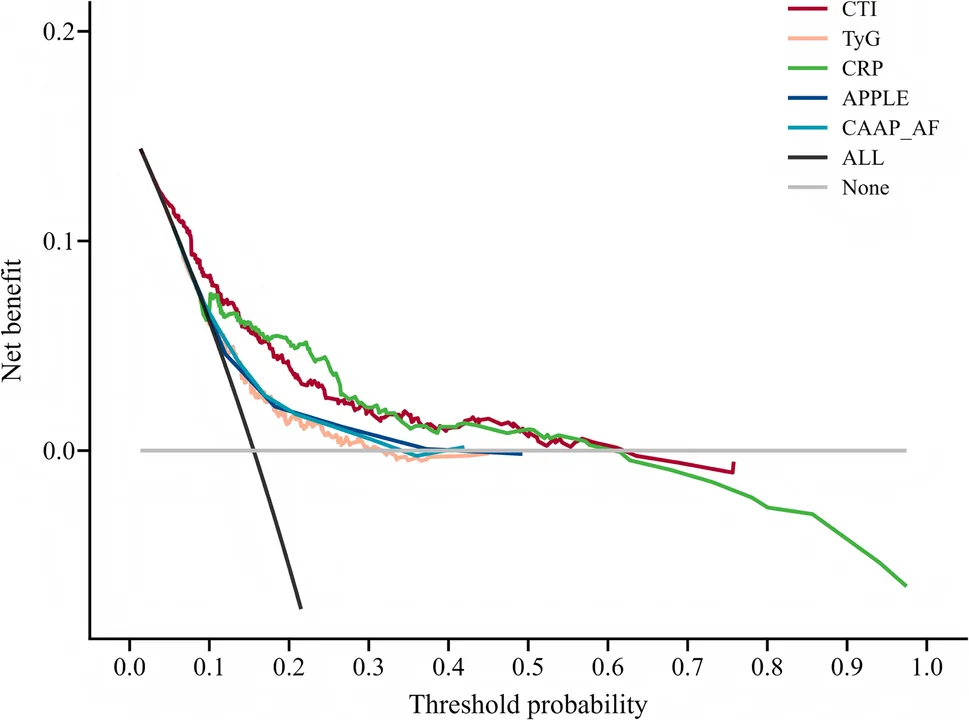
DCA

ROC curve analysis demonstrated that the AUC for CTI, CRP, TyG index, CAAP-AF score and APPLE score in predicting AF recurrence was 0.755 (95% CI: 0.704–0.806), 0.719 (95% CI: 0.654–0.784), 0.633 (95% CI: 0.570–0.696), 0.650(95%CI:0.588,0.712) and 0.643(95%CI:0.580,0.706) respectively (Supplementary Table 2, Fig. 5). All five indices yielded AUC values greater than 0.5, indicating the presence of discriminatory ability. Compared with CRP, TyG index, CAAP-AF score and APPLE score CTI exhibited an AUC with a 95% CI within the range of 0.7 to 0.9, suggesting moderate to good discriminatory performance for identifying AF recurrence risk. To further assess clinical use. Using the DeLong test, we found that the AUC of CTI was significantly higher than that of the TyG index (*p* < 0.001), APPLE score (*p* = 0.007), and CAAP-AF score (*p* = 0.013). Although CTI showed a numerically higher AUC than CRP, the difference was not statistically significant (*p* = 0.080). No significant differences were observed between the other pairwise comparisons of the remaining markers (all *p* > 0.05) (Table 2). DCA was conducted. Within a threshold probability range of 0.0 to 0.70, CTI consistently demonstrated a higher net benefit than both the “treat-all” and “treat-none” strategies, indicating potential clinical value in guiding interventions aimed at reducing AF recurrence. In contrast, CRP, the TyG index, APPLE score, and CAAP-AF score only provided positive net benefit over the reference strategies within much narrower ranges of threshold probabilities; The TyG index, in particular, showed the narrowest range of positive net benefit (approximately 0.05 to 0.30). Similarly, CRP, APPLE, and CAAP-AF also had more limited clinical utility compared with the CTI (Fig. 6). To evaluate the incremental predictive value of the CTI compared with traditional markers, we performed net reclassification NRI and IDI analyses. Compared with single biomarkers and traditional risk scores, the CTI demonstrated superior predictive performance. NRI and IDI analyses revealed that, compared with the TyG index, the APPLE score and the CAAP-AF score, the CTI showed significant improvements in categorical NRI (*p* = 0.0013), continuous NRI (all *p* < 0.0001) and IDI (all *p* < 0.0001). Although no significant differences were observed between CTI and CRP in terms of categorical NRI and IDI, continuous NRI still showed a significant improvement (*p* = 0.0006) (Table 3).

**Table 2:** DeLong test results comparing the predictive performance of CTI with other markers for AF recurrence after RFCA

Contrast	delta_AUC	z	*p* value
CTI vs. TyG	0.122 (0.056 to 0.188)	3.614	< 0.001
CTI vs. CRP	0.036 (-0.004 to 0.076)	1.752	0.080
CTI vs. APPLE	0.112 (0.031 to 0.193)	2.714	0.007
CTI vs. CAAP-AF	0.105 (0.023 to 0.188)	2.495	0.013
CRP vs. TyG	0.086 (-0.013 to 0.184)	1.710	0.087
APPLE vs. TyG	0.010 (-0.078 to 0.097)	0.221	0.825
CAAP_AF vs. TyG	0.017 (-0.074 to 0.107)	0.364	0.716
APPLE vs. CRP	-0.076 (-0.168 to 0.016)	-1.628	0.104
CAAP_AF vs. CRP	-0.069 (-0.162 to 0.024)	-1.462	0.144
CAAP_AF vs. APPLE	0.007 (-0.029 to 0.043)	0.377	0.706

**Table 3:** Comparison of NRI and IDI for CTI versus conventional predictors of AF recurrence

Contrast	AUC	*p* value
CTI vs. TyG		
NRI(Categorical) [95% CI]	0.2076 (0.1084 to 0.3069)	0.0001
NRI(Continuous) [95% CI]	0.7335 (0.5224 to 0.9446)	< 0.0001
IDI [95% CI]	0.0943 (0.0648 to 0.1237)	< 0.0001
CTI vs. CPR		
NRI(Categorical) [95% CI]	0.0380 (-0.0518 to 0.1278)	0.4068
NRI(Continuous) [95% CI]	0.3832 (0.1633 to 0.6032)	0.0006
IDI [95% CI]	0.0172 (-0.0126 to 0.0470)	0.2578
CTI vs. APPLE		
NRI(Categorical) [95% CI]	0.2133 (0.1075 to 0.3191)	0.0001
NRI(Continuous) [95% CI]	0.4582 (0.2406 to 0.6757)	< 0.0001
IDI [95% CI]	0.0835 (0.0447 to 0.1224)	< 0.0001
CTI vs. CAAP-AF		
NRI(Categorical) [95% CI]	0.1427 (0.0148 to 0.2706)	0.0288
NRI(Continuous) [95% CI]	0.4919 (0.2754 to 0.7085)	< 0.0001
DI [95% CI]	0.0831 (0.0423 to 0.1238)	< 0.0001

### Calibration assessment of the CTI

**Fig. 7:**
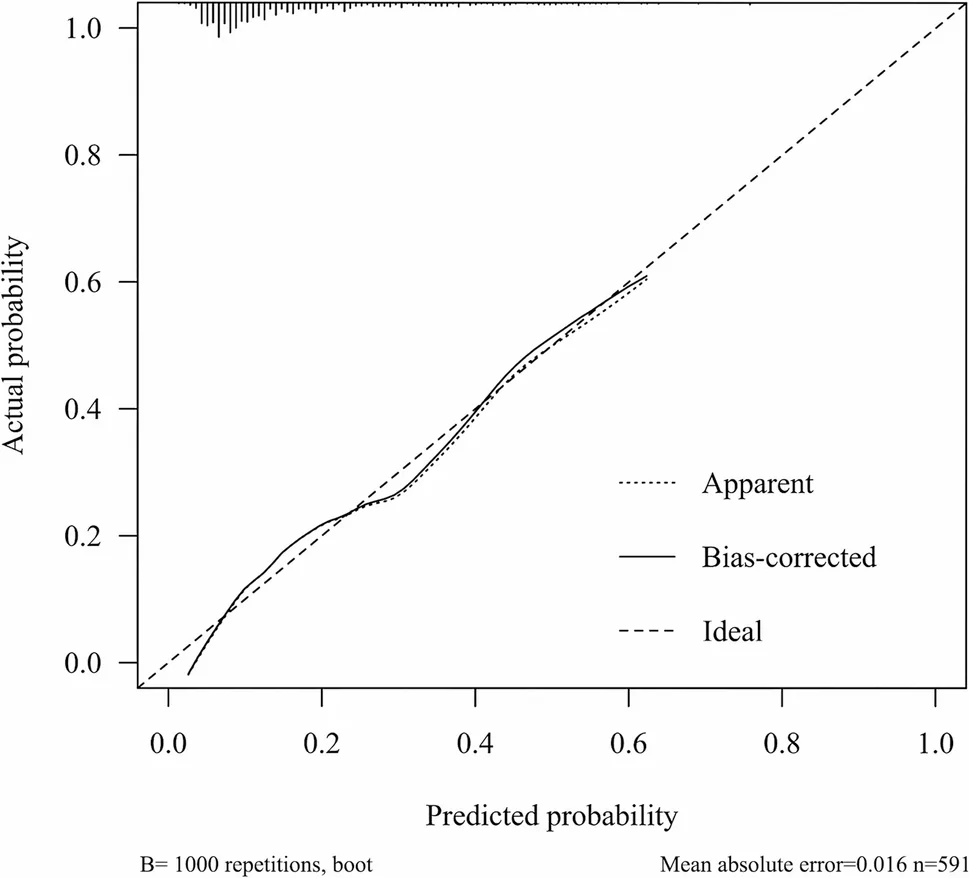
Calibration curve for the CTI-based prediction model of late AF recurrence after RFCA

The calibration of CTI was assessed using the HL test and calibration curves. The HL test indicated no significant difference between the predicted and observed results for the CTI (χ² = 8.014, *p* = 0.432), suggesting that the model is well calibrated (Supplement Table 2). Similarly, the calibration of the CTI for predicting AF recurrence was assessed using a calibration curve with 1000 bootstrap repetitions (*n* = 591). The curve demonstrated excellent agreement between predicted probabilities and observed outcomes, with a mean absolute error of 0.016. The calibration line closely followed the ideal 45° reference line across the range of predicted probabilities, indicating no significant overestimation or underestimation of recurrence risk (Fig. 7). This finding was consistent with the non-significant result of the HL-test confirming adequate calibration of the CTI.

### Subgroup analysis

**Fig. 8:**
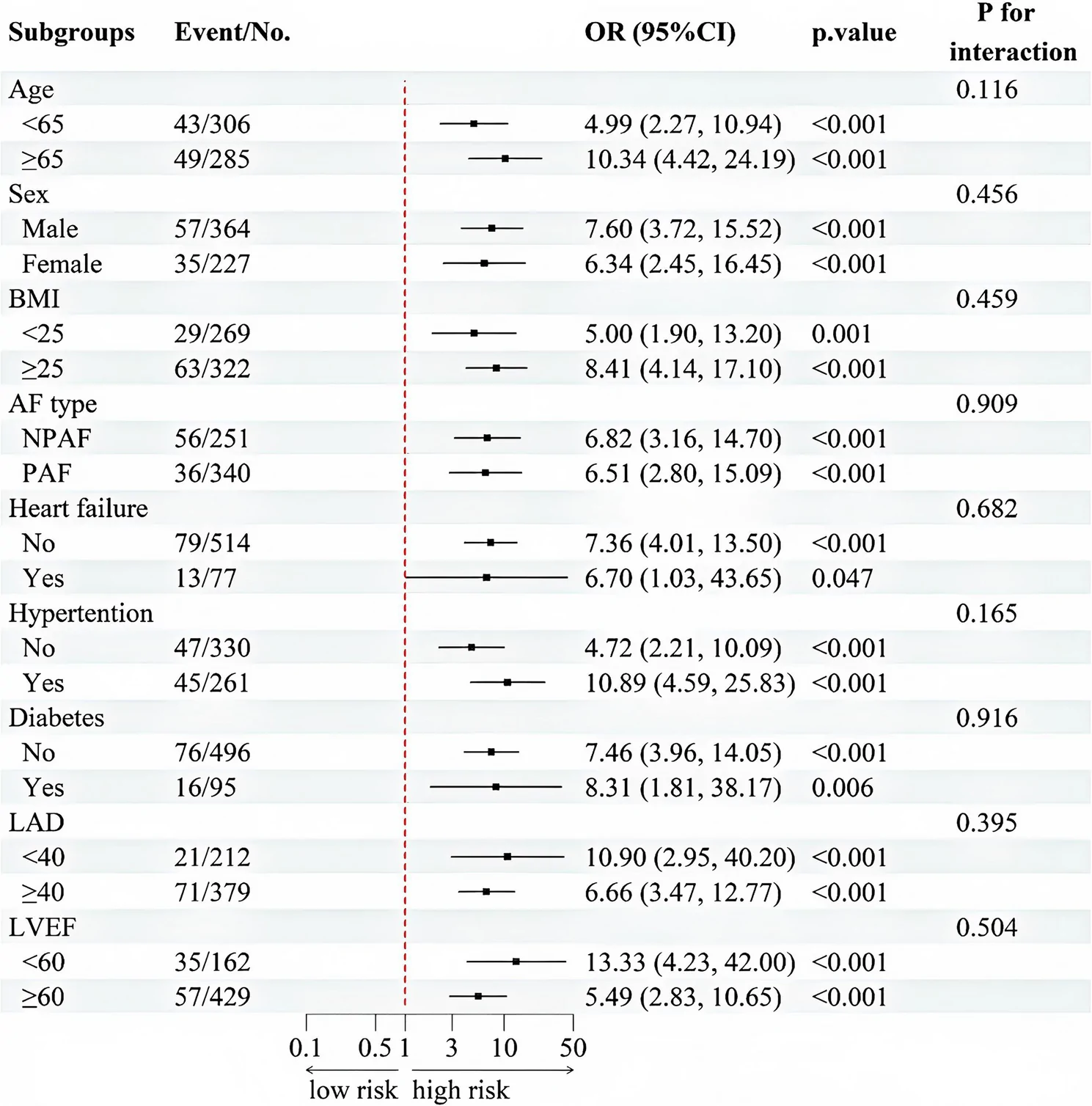
Subgroup analysis of the association between CTI and AF recurrence. (OR, Oddsratio; CI, confidence interval)

Stratified subgroup analyses were performed according to clinical characteristics, including age, sex, BMI, AF type, heart failure, hypertension, DM, LAD, and LVEF. Interaction tests were conducted to assess potential heterogeneity in the strength of the association across subgroups (Fig. 8). CTI was significantly and positively associated with the risk of AF recurrence in all subgroups (all ORs > 1 and all *p* values < 0.05). No reversal in the direction of the association or loss of statistical significance was observed in any subgroup.

All *p* values for interaction were > 0.05, indicating no statistically significant differences in the magnitude of the association between CTI and AF recurrence risk across the assessed subgroups. The ORs and corresponding 95% CIs for each subgroup are presented in Fig. 8.

## Discussion

This study systematically assessed the relationship between CTI and the risk of late AF recurrence after RFCA. The findings demonstrated that each one-unit increase in CTI was associated with an OR of 7.16 for AF recurrence (95% CI: 4.32–11.87), and patients in the third tertile group exhibited a 7.99-fold higher risk of recurrence compared with those in the first tertile group (95% CI: 3.95–16.19). RCS analysis suggested a nonlinear and threshold-like association between CTI and AF recurrence, although the nonlinear test did not reach statistical significance (*P* = 0.072) owing to limited event numbers, supporting the potential use of CTI as a predictor of AF recurrence. These results underscore the clinical relevance of CTI as an integrated marker reflecting IR and inflammatory status, providing additional insight into mechanisms associated with AF recurrence. Meanwhile, we noted an interesting phenomenon in this study: the proportion of patients with prior stroke was significantly higher in the non-recurrence group than in the recurrence group (13.8% vs. 4.3%, *p* = 0.011). On the one hand, this may be attributed to the lower rate of preoperative anticoagulant use in the non-recurrence group (41.3% vs. 54.3%, *p* = 0.02). Insufficient anticoagulation may increase the risk of stroke, which could partly explain the higher prevalence of prior stroke in the non-recurrence group. Furthermore, given the limited sample size in the recurrence group, there is a high likelihood of random imbalances in baseline characteristics between the two groups, and random sampling error may further amplify the difference in the proportion of previous strokes between the two groups. At the same time, selection bias inherent in retrospective observational studies may also have an impact on this.

AF, recognized as a progressive disease continuum. From an etiological perspective, AF represents a complex pathological condition involving multiple alterations at the molecular, cellular, electrophysiological, and structural levels. The diverse mechanisms and pathways underlying AF collectively account for the incomplete elucidation of its pathogenesis [19, 20]. It has been extensively reported to be closely associated with left atrial remodeling and chronic inflammation. IR, characterized by reduced responsiveness of peripheral tissues to insulin, represents a central pathophysiological mechanism underlying metabolic syndrome. It is typically manifested by elevated FBG, TG, fasting insulin levels, and visceral adiposity, together with reduced HDL-C, and is closely linked to oxidative stress, inflammation, and cardiovascular remodeling [21, 22]. IR contributes significantly to the pathogenesis of AF through multiple pathways [23]. As a surrogate marker for IR, the TyG index has been reported to demonstrate superior accuracy compared with the HOMA-IR [24]. Sang et al. further reported that the TyG index was an independent risk factor for early AF recurrence after RFCA. Both AF prevalence and recurrence rates were higher in patients with elevated TyG index values compared with those with lower values, with this association being particularly pronounced among patients with persistent AF [25]. Tang et al. conducted a retrospective study focusing on patients without diabetes undergoing cardiac radiofrequency ablation and further demonstrated that an elevated TyG index was associated with an increased risk of late AF recurrence following the procedure [26]. In contrast, CRP is a well-established biomarker of both local and systemic inflammation. Prior studies conducted by Zhang et al. demonstrated that inflammation plays a key role in the initiation and maintenance of AF [27, 28]. Studies conducted by Meyre et al., Letsas et al., and Liu et al. confirmed a significant association between CRP levels and AF recurrence following radiofrequency ablation, with elevated baseline CRP identified as an independent predictor of recurrence [13, 29, 30].

The CTI, a composite biomarker reflecting both IR and systemic inflammatory status, integrates two established predictive markers; CRP and the TyG index into a single metric. Accumulating evidence has demonstrated its strong predictive value for various adverse outcomes, including CVD, stroke, and cardiovascular–kidney–metabolic (CKM) syndrome events. For example, in a 9-year prospective cohort study, Zhang et al. reported a significant association between CTI and new-onset CVD during follow-up. After comprehensive adjustment for demographic characteristics, lifestyle factors, and clinical risk factors, each 1-unit increase in CTI was associated with a 44% higher risk of CVD, and its predictive performance exceeded that of the TyG index and CRP when assessed individually [31]. Tang et al. further demonstrated in a prospective cohort study that CTI, as a composite indicator, predicted stroke risk in patients with hypertension. Cox regression analysis indicated that each 1-unit increase in CTI was associated with a 21% increased risk of stroke (hazard ratio [HR] = 1.21, 95% CI: 1.08–1.37). The risk of stroke in the highest CTI quartile was 66% higher than that in the lowest quartile (HR = 1.66, 95% CI: 1.23–2.25) [15]. Ying et al. reported that among patients with CKM syndrome, higher CTI levels were associated with significantly increased risks of all-cause mortality (HR = 1.56; 95% CI: 1.36–1.78) and cardiovascular mortality (HR = 2.03; 95% CI: 1.49–2.77) [32]. Currently, no clinical studies have assessed the association between CTI and late AF recurrence after RFCA.

Although the TyG index and CRP have demonstrated independent predictive value, the present study addressed the absence of evidence regarding their combined predictive relevance in this clinical context. An association between CTI and the rate of late AF recurrence after ablation was observed. After adjustment for demographic and clinical risk factors, the association remained statistically significant (OR = 6.23, 95% CI: 3.59–10.83).Subgroup analyses indicated a significant positive association between CTI and AF recurrence risk across all assessed subgroups. This association remained consistent across different population characteristics, with no statistically significant heterogeneity observed, supporting the stability and generalizability of the findings. RCS analysis revealed an overall association between the CTI and AF recurrence after RFCA. Nevertheless, the test for nonlinearity yielded a P value of 0.072, which did not reach the conventional statistical significance threshold and only indicated a trend toward nonlinearity. This non-significant finding may be attributable to limited statistical power due to the small number of outcome events in our cohort. The spline curve showed an increasing trend in AF recurrence risk when CTI exceeded 4.5. This cutoff value cannot be used as a standardized threshold for clinical risk stratification, and merely provides a preliminary exploratory direction for subsequent risk-stratification studies with larger sample sizes. Notably, the composite index CTI demonstrated significantly superior predictive performance compared with the TyG index, APPLE score, and CAAP‑AF score. Although the difference between CTI and CRP did not reach statistical significance, receiver operating characteristic curve analysis yielded an area under the curve of 0.755 (95% CI: 0.704–0.806) for CTI in predicting post‑ablation AF recurrence, which was significantly higher than that of conventional TyG, APPLE, and CAAP‑AF scores, and also showed a favorable trend relative to CRP. These findings suggest that integrating inflammatory and metabolic biomarkers may confer additional predictive value, which was further validated by NRI and IDI analyses. We systematically evaluated the incremental predictive value of CTI relative to established AF recurrence markers. Compared with TyG, CRP, APPLE, and CAAP‑AF scores, the CTI model exhibited consistently better predictive performance, with significantly positive continuous NRI and IDI values across all comparisons (*p* < 0.0001). This indicates that CTI substantially improves risk reclassification and enhances overall discriminative ability. Such an integrative approach effectively improves predictive accuracy and stability, overcoming the limitation of single‑marker assessments with one‑dimensional evaluation.

This integrative analytical approach enhanced the identification and stratification of patients at elevated risk. Compared with the TyG index or CRP alone, CTI incorporates both markers, thereby reflecting metabolic and inflammatory pathways simultaneously and enabling a more comprehensive evaluation of metabolic–inflammatory status. In contrast, individual biomarkers represent a single dimension of the underlying pathophysiological process, whereas their combined assessment facilitates evaluation of potential interactive effects and enables comprehensive assessment of the synergistic roles of metabolic and inflammatory pathways in AF recurrence. Given substantial inter-individual variability in biomarker levels, the TyG index and CRP provide complementary biological data regarding IR and inflammatory status, respectively [33].

Although CTI demonstrated potential predictive value for late AF recurrence after radiofrequency ablation, the underlying mechanisms have not been fully elucidated. From prior studies, this association may be attributable to atrial remodeling induced by IR through multiple pathways, including increased oxidative stress and enhanced nicotinamide adenine dinucleotide phosphate oxidase activity; disruption of intracellular calcium homeostasis mediated by oxidation of calcium/calmodulin-dependent protein kinase II and phosphorylation of the ryanodine receptor; increased collagen deposition and upregulation of transforming growth factor-β1, promoting atrial structural remodeling through paracrine and autocrine signaling pathways and thereby increasing susceptibility to AF; activation of the mitogen-activated protein kinase signaling pathway, contributing to atrial electrical and structural remodeling [34, 35]; and the interrelationship between IR and oxidative stress–related injury, leading to left atrial fibrosis and the development of left atrial low-voltage areas.

Reduced glucose metabolic capacity in atrial tissue among patients with IR has been reported to result in excessive intracellular glucose accumulation and the formation of advanced glycation end products (AGEs). AGEs promote both local and systemic inflammatory responses, thereby impairing atrial electrophysiological and structural properties and contributing to irreversible atrial structural and electrical remodeling [8, 36]. Inflammation has been recognized as a central contributor to atrial cardiomyopathy. Chronic inflammatory activation mediated by cytokines, including interleukin-6, tumor necrosis factor-α, and CRP, has been associated with atrial fibrosis, changes in intercellular junction proteins, and persistence of AF [37–39]. Therefore, inflammation plays a key role in the initiation and maintenance of AF.

Studies by Chen et al. further demonstrated inflammatory features in atrial tissue samples obtained from patients with AF, including fibrosis, leukocyte infiltration, and oxidative injury. These inflammatory changes may contribute to atrial electrical and structural remodeling, thereby increasing susceptibility to AF recurrence [40]. Chronic low-grade inflammation has been implicated in the pathogenesis of IR through activation of nuclear factor-κB, c-Jun N-terminal kinase, Wnt, and Toll-like receptor signaling pathways [41]. The interaction among these mechanisms may create a self-perpetuating cycle that contributes to AF recurrence following radiofrequency ablation.

The findings of this study support the potential clinical use of CTI as a biomarker for predicting recurrence risk in patients with AF undergoing raRFCA. A significant positive association was observed between CTI levels and late postoperative AF recurrence, with patients in the third tertile demonstrating a markedly higher recurrence risk compared with those in the first tertile. These observations underscore the potential importance of routine preprocedural assessment of CRP and the TyG index, together with CTI-based risk stratification. In patients with CTI values exceeding 4.5, early postoperative lifestyle interventions, including appropriate exercise, dietary adjustment and weight management, may be beneficial for reducing AF recurrence. The potential benefits of insulin-sensitizing agents such as SGLT-2 inhibitors and low-dose anti-inflammatory strategies such as colchicine in this population remain to be validated in future high-quality randomized controlled trials. Rigorously designed trials are warranted to determine whether metabolic or anti-inflammatory interventions targeted at patients with elevated CTI can lower the risk of postoperative AF recurrence.

### Limitations

Several limitations should be considered. First, as a retrospective cohort study, the possibility of selection bias could not be entirely excluded. Second, CTI was assessed only at baseline (upon admission) and was not dynamically monitored over time; therefore, temporal changes particularly in the preoperative and postoperative periods were not assessed. Dynamic assessment may allow characterization of intra-individual trajectories and reduce potential regression dilution bias. Third, the study population was derived from a single center within one geographic region and consisted of individuals of the same ethnicity. The generalizability of the findings may be limited, and extrapolation to populations from other regions or ethnic backgrounds should be undertaken with caution due to potential referral bias and related factors. Fourth, the multivariate regression model included in this study utilized 11 adjusting covariates, while the number of endpoint events of atrial fibrillation recurrence was only 92. The number of cases for each variable was limited, raising the risk of model overfitting. Fifth, as a single-center retrospective study, we did not perform a stratified analysis according to different ablation strategies. Although most of patients with persistent AF underwent a standardized ablation protocol in our center, including mitral isthmus linear ablation, left atrial roof line ablation, and ethanol infusion of the vein of Marshall, and our modified mitral isthmus ablation achieved a high bidirectional block rate of 98.3% [42], which partially mitigated confounding from procedural variations, residual confounding related to unmeasured procedural factors cannot be fully excluded. Futhermore, this observational study demonstrated an association between CTI and AF recurrence but did not examine underlying mechanisms or assess whether reduction in CTI through lifestyle modification, insulin-sensitizing therapy, or anti-inflammatory treatment could decrease post-ablation AF recurrence. Therefore, future prospective, multicenter studies with longer follow-up durations are warranted to validate these findings and to further examine potential causal mechanisms.

## Conclusions

Elevated CTI levels, a composite biomarker reflecting both IR and systemic inflammation, were significantly associated with an increased risk of AF recurrence following RFCA. These findings support the potential utility of CTI in risk stratification for AF recurrence. In addition, therapeutic strategies targeting IR and inflammatory pathways may represent potential approaches for the comprehensive management of AF in clinical practice.

## Supplementary Information

Supplementary Material 1.

Supplementary Material 2.

## Data Availability

The datasets used and/or analyzed during the current study are available from the corresponding author upon reasonable request.

## Abbreviations

AF: Atrial fibrillation
RFCA: Radiofrequency Catheter Ablation
IR: Insulin Resistance
HOMA-IR: Homeostasis model assessment of Insulin Resistance
TyG: Triglyceride-Glucose
CRP: C-reactive protein
CVD: Cardiovascular Disease
CTI: C-reactive Protein-Triglyceride glucose Index
NVAF: Non-Valvular Atrial Fibrillation
BMI: Body Mass Index
SGLT-2: Sodium-Glucose Cotransporter 2
ARNI: Angiotensin Receptor–Neprilysin Inhibitors
FBG: Fasting Blood Glucose
TG: triglycerides
TC: Total Cholesterol
LDL-C: Low-Density Lipoprotein Cholesterol
HDL-C: High-Density Lipoprotein Cholesterol
HGB: hemoglobin
NT-proBNP: N-terminal pro-B-type natriuretic peptide
LAD: Left Atrial Diameter
LVEF: Left Ventricular Ejection Fraction
DM: Diabetes Mellitus
CHD: Coronary Heart Disease
CARTO: Three-Dimensional Electroanatomical Mapping System
CPVI: Circular Pulmonary Vein Isolation
PAF: Paroxysmal Atrial Fibrillation
NPAF: Non-Paroxysmal Atrial Fibrillation
AADs: Antiarrhythmic Drugs
ECG: Electrocardiogram
AT: Atrial Tachycardia
SD: Mean ± Standard Deviation
ANOVA: Analysis of Variance
RCS: Restricted cubic spline
ROC: Receiver Operating Characteristic
AUC: Area Under the Curve
CI: Confidence Interval
DCA: Decision Curve Analysis
OR: Odds Ratio
CKM: Cardiovascular-Kidney-Metabolic Syndrome
HR: Hazard Ratio
AGEs: Advanced Glycation End Products
NRI: Net Reclassification Improvement
IDI: Integrated Discrimination Improvement
HL test: Hosmer–Lemeshow (HL) test
