# Supplementary material for: Predictive value of the C-reactive protein–triglyceride–glucose index for late atrial fibrillation recurrence after radiofrequency catheter ablation: a retrospective cohort study
Source: BMC Cardiovasc Disord. 2026 Jul 22;26:855. doi: 10.1186/s12872-026-06238-1 (PMC13628736; doi:10.1186/s12872-026-06238-1)
Supplement: Supplementary file 1 [file 12872_2026_6238_MOESM1_ESM.docx]

**Supplementary Table 1.** Baseline Characteristics of Patients Stratified by Tertiles of CTI

| Characteristics | Total (n = 591) | Q1 (n = 197) | Q2 (n = 197) | Q3 (n = 197) | p value |
| --- | --- | --- | --- | --- | --- |
| **Demographics** |  |  |  |  |  |
| Male, n(%) | 364 (61.6) | 127 (64.5) | 109 (55.3) | 128 (65) | 0.086 |
| Age, years, Mean ± SD | 62.55 ± 10.57 | 63.18 ± 9.96 | 63.02 ± 10.05 | 61.46 ± 11.59 | 0.237 |
| BMI, kg/m^2^, Mean ± SD | 25.66 ± 3.85 | 25.28 ± 3.43 | 25.55 ± 3.68 | 26.13 ± 4.37 | 0.100 |
| **Medical history** |  |  |  |  |  |
| Hypertension, n(%) | 261 (44.2) | 76 (38.6) | 95 (48.2) | 90 (45.7) | 0.136 |
| Diabetes mellitus (%) | 95 (16.1) | 21 (10.7) | 29 (14.7) | 45 (22.8) | 0.004 |
| Coronary heart disease (%) | 65 (11) | 30 (15.2) | 17 (8.6) | 18 (9.1) | 0.066 |
| Congestive heart failure, n(%) | 77 (13) | 25 (12.7) | 18 (9.1) | 34 (17.3) | 0.056 |
| Stroke (%) | 73 (12.4) | 18 (9.1) | 32 (16.2) | 23 (11.7) | 0.094 |
| **Laboratory data** |  |  |  |  | 0.136 |
| BNP, pg/mL, Median (IQR) | 436 (148 - 1010) | 427 (170 - 946) | 411.00 (123 - 966) | 487.00 (146 - 1130) | 0.372 |
| Cr, μmol/L, Median (IQR) | 67.00 (56.00 - 78.65) | 67.00 (57.00 - 78.00) | 65 (55.00 - 78.80) | 67.00 (58.00 - 78.00) | 0.509 |
| TC, mmol/L, Median (IQR) | 3.88 (3.28 - 4.50) | 3.70 (3.14 - 4.27) | 4.04 (3.36 - 4.67) | 3.86 (3.32 - 4.64) | 0.003 |
| LDL, mmol/L, Median (IQR) | 2.16 (1.66 - 2.65) | 2.05 (1.56 - 2.51) | 2.23 (1.71 - 2.76) | 2.23 (1.73 - 2.72) | 0.021 |
| HDL, mmol/L, Median (IQR) | 1.08 (0.93 - 1.28) | 1.19 (0.99 - 1.37) | 1.08 (0.98 - 1.26) | 1.00 (0.84 - 1.16) | < 0.001 |
| HGB, g/L, Mean ± SD | 139.37 ± 16.99 | 139.75 ± 13.68 | 138.71 ± 17.57 | 139.66 ± 19.30 | 0.794 |
| FBG, mg/dl, Mean ± SD | 107.90 ± 31.66 | 97.39 ± 20.96 | 106.80 ± 24.98 | 119.49 ± 41.32 | < 0.001 |
| TG, mg/dL, Mean ± SD | 129.69 ± 73.29 | 87.03 ± 23.47 | 130.10 ± 46.82 | 171.95 ± 99.01 | < 0.001 |
| TyG, Mean ± SD | 8.70 ± 0.55 | 8.29 ± 0.35 | 8.76 ± 0.38 | 9.06 ± 0.58 | < 0.001 |
| CRP, mg/L, Mean ± SD | 1.29 ± 1.49 | 0.48 ± 0.18 | 0.75 ± 0.46 | 2.63 ± 1.92 | < 0.001 |
| CTI, Median ± SD | 4.63 ± 0.46 | 4.17 ± 0.15 | 4.55 ± 0.11 | 5.17 ± 0.31 | < 0.001 |
| **UCG** |  |  |  |  |  |
| LAD, Median (IQR) | 42 (36 - 46) | 41 (36 - 45) | 42 (35 - 46) | 42 (37 - 46) | 0.128 |
| LVEF, Median (IQR) | 63 (59 - 67) | 62(59- 66) | 64 (60 - 67) | 62(58 - 67) | 0.040 |
| **Drug history** |  |  |  |  |  |
| amiodarone | 31 (5.2) | 11 (5.6) | 9 (4.6) | 11 (5.6) | 0.873 |
| ARNI (%) | 82 (13.9) | 20 (10.2) | 29 (14.7) | 33 (16.8) | 0.152 |
| SGLT2 (%) | 44 (7.4) | 6 (3) | 12 (6.1) | 26 (13.2) | < 0.001 |
