# Supplementary material for: Predictive value of the C-reactive protein–triglyceride–glucose index for late atrial fibrillation recurrence after radiofrequency catheter ablation: a retrospective cohort study
Source: BMC Cardiovasc Disord. 2026 Jul 22;26:855. doi: 10.1186/s12872-026-06238-1 (PMC13628736; doi:10.1186/s12872-026-06238-1)
Supplement: Supplementary file 2 [file 12872_2026_6238_MOESM2_ESM.docx]

**Supplementary Table 2.** Diagnostic performance of five indicators for predicting AF recurrence after RFCA

| Characteristics | CTI | TyG | CRP | APPLE | CAAP_AF |
| --- | --- | --- | --- | --- | --- |
| Cut off | 4.654 | 8.735 | 2.165 | 1.500 | 5.500 |
| AUC | 0.755(0.704, 0.806) | 0.633(0.570, 0.696) | 0.719(0.654, 0.784) | 0.643(0.580, 0.706) | 0.650(0.588, 0.712) |
| p value | <0.001 | <0.001 | <0.001 | <0.001 | <0.001 |
| Sens | 0.728(0.637, 0.819) | 0.620(0.520, 0.719) | 0.522(0.420, 0.624) | 0.641(0.543, 0.739) | 0.489(0.387, 0.591) |
| Spec | 0.657(0.616, 0.699) | 0.595(0.552, 0.638) | 0.868(0.838, 0.897) | 0.581(0.538, 0.624) | 0.735(0.697, 0.774) |
| Accuracy rate | 0.668(0.668, 0.669) | 0.599(0.598, 0.600) | 0.814(0.813, 0.814) | 0.591(0.590, 0.591) | 0.697(0.696, 0.698) |
| PPV | 0.282(0.224, 0.339) | 0.220(0.170, 0.271) | 0.421(0.330, 0.512) | 0.220(0.171, 0.270) | 0.254(0.190, 0.318) |
| NPV | 0.929(0.902, 0.956) | 0.895(0.862, 0.928) | 0.908(0.882, 0.934) | 0.898(0.865, 0.931) | 0.886(0.856, 0.917) |
| KAPPA | 0.234(0.165, 0.303) | 0.123(0.059, 0.188) | 0.355(0.259, 0.451) | 0.125(0.062, 0.188) | 0.163(0.082, 0.244) |
| Jorden Index | 0.385 | 0.215 | 0.390 | 0.222 | 0.224 |
| PLR | 2.125(1.785, 2.530) | 1.531(1.263, 1.855) | 3.945(2.928, 5.314) | 1.531(1.273, 1.841) | 1.849(1.433, 2.386) |
| NLR | 0.413(0.294, 0.581) | 0.639(0.488, 0.838) | 0.551(0.444, 0.684) | 0.617(0.465, 0.819) | 0.695(0.565, 0.854) |
| HL-test | X^2^=8.014; p=0.432 | X^2^=6.213; p=0.623 | X^2^=10.152; p=0.180 | X^2^=1.342; p=0.511 | X^2^=NaN; p=NaN |

Sens: Sensitivity; Spec: Specificity; PPV: Positive predictive value; NPV:N egative predictive value; HL-Test: Hosmer-Lemeshow Test
